# Noise-Induced Hearing Threshold Shift Correlated with Body Weight and External-Ear Amplification in Chinchilla: a Preliminary Analysis

**DOI:** 10.1007/s10162-023-00913-2

**Published:** 2023-11-27

**Authors:** Sarah K. Grinn, Monica Trevino, Edward Lobarinas

**Affiliations:** 1 College of Health Professions, Central Michigan University, Mount Pleasant, MI USA; 2 School of Behavioral and Brain Sciences, The University of Texas at Dallas, Dallas, TX USA

**Keywords:** Noise-exposure, Hearing loss susceptibility, External-ear amplification, Auditory brainstem response

## Abstract

**Background:**

External-ear amplification (EEA) has been shown to vary from 5–19 dB-A in large datasets of pediatric, adolescent, and adult human participants. However, variable EEA is an overlooked characteristic that likely plays a role in individual noise-induced hearing loss (NIHL) susceptibility. A noise exposure varying 5–19 dB-A translates to high-EEA individuals theoretically experiencing 3–4 times greater NIHL risk than low-EEA individuals.

**Objective:**

The purpose of this preliminary analysis was to test the hypothesis that higher EEA is correlated with increased noise-induced threshold shift susceptibility.

**Design:**

Nine chinchillas were exposed to 4-kHz octave-band noise at 89 dB-SPL for 24 h. Auditory brainstem response thresholds were obtained pre-exposure, 24-h post-exposure, and 4-week post-exposure. Relationships between EEA and threshold shift were analyzed.

**Results:**

Open-ear EEA ranged 11–19 dB-SPL, and occluded-ear EEA ranged 10–21 dB-SPL. Higher occluded-ear EEA was correlated with increased NIHL susceptibility (*p* = 0.04), as was lower body weight (*p* = 0.01). Male animals exhibited more threshold shift than female animals (*p* = 0.02), lower body weight than female animals (*p* = 0.02), and higher occluded-ear EEA (male mean = 18 dB; female mean = 15 dB).

**Conclusions:**

Taken together, increased threshold shift susceptibility was observed in the smallest animals, animals with the highest occluded-ear EEA, and in male animals (which tended to have higher occluded-ear EEA). Given the established relationship between smaller body size and higher occluded-ear EEA, these preliminary results suggest that body size (and occluded-ear EEA; a function of body size) could be a potential, underlying driver of NIHL susceptibility differences, rather than true sex differences.

## Introduction

In humans, there is significant variability in susceptibility to noise-induced hearing loss (NIHL) across individuals, even when exposed to the same or similar degrees of noise hazard [1–5]. Data from the International Standards Organization (ISO) 1999:2013 (2013) showed variable hearing loss ranging from 14 to 33 dB HL in workers with careers of a similar 100 dB-A occupational noise exposure. In a prospective experimental study of young adults exposed to 100 dB-SPL of headphone music for four hours [6], temporary threshold shift (TTS) ranged from − 5 to 14 dB HL at 15-min post-exposure testing, thereby confirming the presence of significant, individual variability in risk of NIHL in a controlled study exposure.

Remarkably, efforts to relate anatomical external-ear differences have not been a part of the risk assessment of NIHL, even though external-ear amplification differences are well-documented [7–22]. Multiple reports have shown that there can be major differences in the external-ear amplification of infants relative to adults [23], and it has long been established that variable head and ear size among adults alone suggest that significant differences in external-ear amplification are present across the adult population [24]. Holding duration of a noise exposure constant, the risk of NIHL doubles with every 3 dB-A increase in exposure level (NIOSH, 1998) [25] or 5 dB-A increase in noise exposure (OSHA, 1983) [26]; therefore, it is likely that external-ear amplification differences of up to 14 dB between two individuals [15] play a significant role in individual susceptibility to NIHL.

Experiments in animals also show significant variability to NIHL susceptibility despite strictly controlled study designs, reduced biological and environmental variability, and careful adherence to experimental protocols. Among animal studies, one of the most established models of NIHL is the chinchilla. This model has been used repeatedly to study NIHL because of the similarities between human and chinchilla hearing. Chinchilla hearing sensitivity, frequency range, and cochlear anatomy closely correlates to that of humans [27]. However, the chinchilla demonstrates higher susceptibility to NIHL relative to other species [28, 29]. Despite this relative susceptibility, the chinchilla has been a reliable and robust model for NIHL. For example, studies observing the anatomical and threshold effects of impulse noise on chinchillas found that whereas some animals experienced no permanent threshold shift (PTS), other animals demonstrated threshold shifts as high as 40 dB [30]. This variability suggests that other factors, such as EEA, could play a role in NIHL. A major study that integrated multiple data sets to create a relationship between temporary threshold shift (TTS) and PTS from over 900 noise-exposed chinchillas found PTS variability as high as 60 dB across animals, independent of differences in experimental procedures [31–35]. Although these studies were not designed to focus on PTS differences among animals, it is clear that individual susceptibility to NIHL critically impacts overall hearing outcomes following noise exposure. Thus far, individual variability has been considered a byproduct of animal noise exposure experiments that parallels human correlates, but has not been explicitly investigated.

To date, no single factor has sufficiently explained NIHL variability across individuals who experience similar exposure to noise. This gap in our understanding of NIHL susceptibility makes it difficult to create comprehensive public health guidelines and regulations for NIHL prevention. Existing Occupational Health and Safety Administration (OSHA) guidelines on NIHL can fall short of adequately protecting up to 25% of the workforce population [36]. These risk estimates are based on only two variables: (1) noise exposure duration and (2) noise exposure sound level. Current NIHL risk guidelines do not take into account any individual differences, such as external-ear amplification (EEA). Undoubtedly, genetics plays some role in NIHL susceptibility, as genetically modified mice have demonstrated higher susceptibility to NIHL than wildtype controls [37, 38]. In humans, however, research in this area has been limited, although researchers have identified as many as 34 gene variants associated with NIHL [39]. Whereas genetic testing may eventually become an effective predictor of NIHL susceptibility, EEA could be used as an immediate, efficient, cost-effective, and direct mean of predicting individual susceptibility to NIHL.

EEA refers to sound amplification contributed by external-ear structures (e.g., pinna, concha, and ear canal) as sound waves encounter them. Estimates of EEA can be derived from a probe microphone placed near (within 2–5 mm) the eardrum. These in-ear probe measurements consistently reveal higher and more accurate sound levels of a noise exposure than those measured outside the ear in the free field. For example, the sound level of a live music concert measured with a sound level meter held near two concert attendee’s ears standing in the same location might reflect similar dB-SPL, but may differ by as much as 5–19 dB-A if measured at the level of the eardrum, according to recent findings in EEA variability across children, adolescents, and adults [14, 15].

NIOSH scientific guidelines for hearing conservation are more conservative than OSHA federal guidelines. NIOSH promotes a lower, daily permissible noise exposure limit and estimates NIHL risk using a 3-dB exchange rate. This means that the noise dose (i.e., quantified estimation of NIHL risk) of a given exposure doubles with each 3-dB increase. Grinn and Le Prell [15] recently showed that individual EEA can differ by 14 dB (range 5–19 dB) between individuals in a dataset of pediatric, adolescent, and adult participants. If we hypothetically explore the difference in noise dose between two individuals exposed to free field noise at 90 dB for two hours, given an EEA difference of 14 dB between the two of them, one individual would hypothetically accrue 79% in-ear noise dose (i.e., 95 dB for 2 h), and the other individual would theoretically accrue a 2.016% in-ear noise dose (i.e., 104 dB for 2 h). Although the two individuals in this hypothetical scenario were exposed to the exact same free field sound level and duration of noise exposure, EEA differences would have profound effects on their individual noise doses, and thus, their susceptibility to NIHL.

The contributions of EEA are well-established in the study of hearing and hearing loss [40]. In fact, in-ear probe microphone measurement of EEA has been the “gold standard” of clinical hearing aid verification (real-ear probe microphone spectrum measurement analysis) since the early 1980s [18, 41–43]. However, the relationship between EEA and NIHL risk has been largely overlooked. Current NIHL regulations and guidelines (OSHA, 1983 and NIOSH, 1998) do not provide for individual differences in EEA when setting permissible or recommended noise exposure limits. Despite the lack of adoption by OSHA and NIOSH, a link between EEA and individual NIHL risk from free field exposures has been suggested in at least one major study [44].

If EEA accounts for a significant portion of individual variability to NIHL, EEA measurements (or proxy measurements) could be used to successfully and pragmatically identify high-risk individuals (i.e., individuals with high-EEA). It is possible to imagine that individuals with high-EEA may be incurring risk of NIHL at exposure levels that are currently considered to be “permissible” or “safe” by OSHA and NIOSH (in other words, these may be the “tender” ears). To evaluate the relationship between EEA and NIHL, we developed an animal model based on the chinchilla. We hypothesized that animals with higher EEA would exhibit more severe temporary threshold shift (TTS) and permanent threshold shift (PTS) following a 24-h laboratory noise exposure. We hypothesized that EEA measurements alone could account for a significant portion of individual differences in NIHL susceptibility.

## Materials and Methods

### Animals

Nine adult (4–5 years) chinchillas (5 male and 4 female) were randomly selected from a larger cohort for this study. Animals were housed individually with free access to food and water in a quiet (ambient noise level < 63 dB-SPL) room on a 12-h light/dark cycle. Animals were actively enrolled in an environmental enrichment program. The experimental procedures and tests performed in this study were approved by The University of Texas at Dallas (UTD) and The University of Texas Southwestern Medical Center (UTSW) Institutional Animal Care and Use Committees.

### Procedures

All ear/auditory measurements were conducted unilaterally (left ear). Awake animals underwent external-ear examination, tympanometry, and body weight measurement. Animals were then sedated (by body weight; ketamine, 40 mg/kg, and xylazine 2 mg/kg, subcutaneous injection (SC) to obtain baseline auditory brainstem response (ABR) threshold measurements and two types of external-ear amplification measurements: (1) real-ear-to-coupler difference measurement (RECD) and real-ear unaided gain measurement (REUG). One week post-baseline threshold assessment, awake animals underwent a calibrated (+ / − 2 dB) free field noise exposure (89 dB-SPL, 4 kHz centered octave-band noise encompassing 2840–5680 Hz with 3 dB slope rolloff) in their home cages with free access to food and water for 24 h. Post-exposure, animals were sedated and ABR measurements were repeated at 24 h and at 4-week post-exposure.

### Otoscopy

Visual examination of the ear canal and tympanic membrane was conducted to assure normal anatomy.

### Tympanometry

A Grason Stadler Instruments Tympstar in compliance with ANSI S3.39 and IEC 601–1 criteria was used to assess ear canal volume (cc), middle ear pressure (daPa), and immittance (ml). Tympanometry was performed prior to 24-h post-exposure ABR testing. A 226 Hz tympanogram was performed and repeated on each animal’s left ear; the repeated results of which were averaged for all subsequent analyses.

#### ABR

ABR thresholds were assessed as a measurement of animal hearing sensitivity at 1, 2, 4, 8, 12, and 16 kHz using Intelligent Hearing Systems (IHS, Miami, FL, USA) hardware and IHS Smart EP software. This system is restricted to animal use and was calibrated by the manufacturer specifically for use in chinchillas. Animals were sedated using a combination of ketamine (40 mg/kg, SC) and xylazine (2 mg/kg, SC). Pulse oximetry and heart rate (Sp02%; bpm) were monitored at 15-min intervals, and animal body temperature was regulated via warming pad throughout testing. Ipsilateral responses were elicited by tone burst stimuli (10 ms duration; 0.5 ms rise/fall; alternating polarity; 21.1/sec) delivered through E-A-RTONE 3A insert earphones and recorded via subdermal needle electrodes. Animal thresholds were defined as the lowest intensity level that reliably and repeatedly produced Wave-V morphology. Following data collection, atipamezole was used to reverse anesthesia (1 mg/kg, SC). Three ABR threshold averages were calculated using the following three pure tone average (PTA) methods: PTA all frequencies (“PTA-all”: 1, 2, 4, 8, 12, and 16 kHz), PTA mid frequencies (“PTA-mid”: 2, 4, and 8 kHz), and PTA high frequencies (“PTA-high”: 8, 12, and 16 kHz).

### External-Ear Amplification (EEA) Measurements

Two measurements of EEA were taken: (1) RECD and (2) REUG (described independently below). All EAA measurements were obtained under anesthesia prior to ABR 24-h post-exposure testing. EEA measurements were performed inside a custom, double-walled sound attenuating sound booth. EEA measurements were performed with the Audioscan Verifit 2 (Dorchester, ON, Canada) real-ear probe microphone system, which measures inside-ear and outside-ear sound levels in the frequency range 0.20 to 12.5 kHz (measured in 1/12 octave-bands).

### EEA Measurement #1: Real-Ear-Unaided-Gain (REUG)

**Fig. 1:**
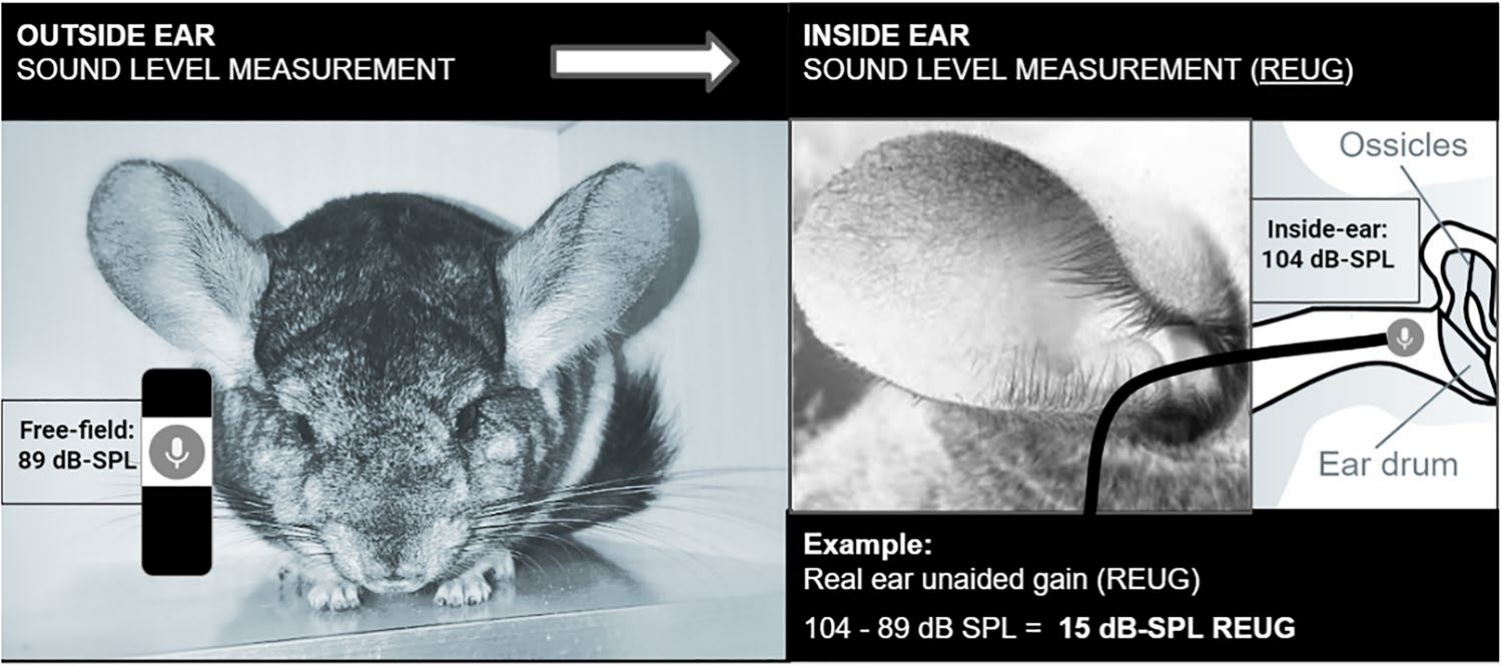
Illustration of the real-ear unaided gain (REUG) measurement. In this example, subject 202 experiences 15 dB-SPL of amplification (104–89 dB-SPL) from the structures of the open-ear (REUG = 15 dB-SPL). Images labeled for reuse with modification: https://www.pexels.com/photo/2168240/; https://www.needpix.com/photo/836334/icon-microphone-voice-message-speak-cloud-data-online-internet; https://commons.wikimedia.org/wiki/File:Chinchilla-ear.JPG

A REUG measurement is a type of EEA measurement. REUG reflects the total amount of gain (amplification) accumulated as a function of passing through the open-ear external structures, each of which boost sound wave amplification (e.g., pinna, concha, and ear canal). As such, REUG is the difference between the sound level observed inside the ear (via in-ear probe microphone, located 2–3 mm from the tympanic membrane) and the sound level observed outside the ear (via reference microphone, located inferior to the lobule). Anesthetized animals were placed facing the free field speaker (the same speaker used to generate the experimental noise exposure), leaving six inches between the speaker and the animal’s nose. First, REUG was measured using a brief sample (12 s) of the exposure stimulus (89 dB-SPL; 4 kHz OBN) generated by RPvdsEx software (Tucker Davis Technologies, Alachua, FL). Speaker amplitude output was calibrated independently for each animal, such that exactly 89 dB-SPL was recorded in the free field by the reference microphone located inferior to the animal’s left pinna (Fig. 1). One can perform this type of sound level measurement in the reference microphone by using the “manual control” sound level measurement function in the Verifit 2 probe microphone measurement system (Dorchester, ON). Once the free field speaker stimulus was adjusted to assure that exactly 89 dB-SPL was measured at the animal’s pinna, a probe microphone was placed inside the sedated animal’s ear canal, approximately 2–3 mm from the eardrum, confirmed by otoscopic examination. This “constant insertion” probe microphone measurement method was used in the human versions of the current study analyzing EEA variability [14, 15]. The constant insertion method has been widely used and validated [45–48]. After the probe microphone was correctly placed, 12 s of the 89 dB-SPL noise exposure stimulus was measured inside the sedated animal’s ear (2–3 mm from the tympanic membrane) using the probe microphone. The inside-ear sound level was measured in 73 frequency bands (1/12th octave analysis) ranging 0.20–12.5 kHz using Fourier transform performed by the Verifit 2 software and extracted via a custom Excel export template, provided by Audioscan. On average, overall sound level (i.e., averaged over a 12 s recording) of the probe microphone’s recording of the noise exposure was calculated. The difference between the sound level measured outside the ear (89 dB-SPL; consistent across all animals) and the sound level measured inside the ear (variable across all animals; due to individual differences in EEA) revealed the animal’s total REUG (Fig. 1).

### EEA Measurement #2: Real-Ear-to-Coupler-Difference (RECD)

**Fig. 2:**
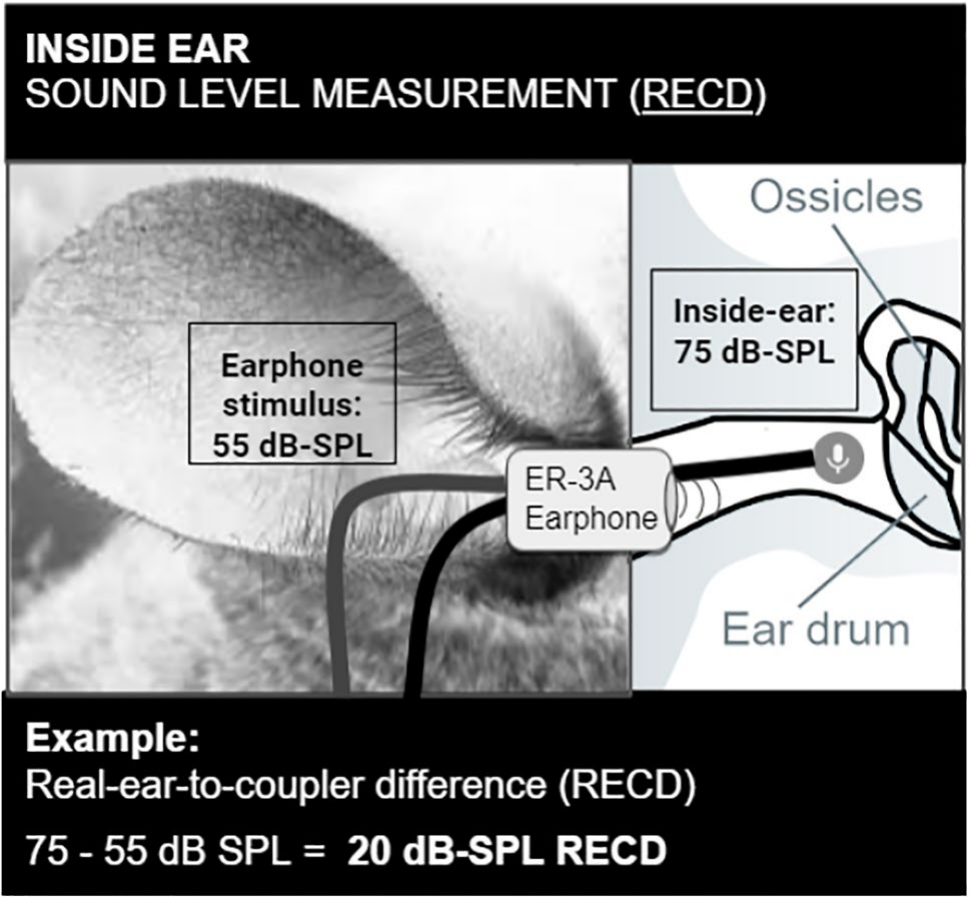
Illustration of the real-ear-to-coupler-difference (RECD) measurement. In this example, subject 202 experiences 20 dB-SPL of amplification (75–55 dB-SPL) from the structure of the ear canal (RECD = 20 dB-SPL). Images labeled for reuse with modification: https://www.needpix.com/photo/836334/icon-microphone-voice-message-speak-cloud-data-online-internet; https://commons.wikimedia.org/wiki/File:Chinchilla-ear.JPG

Following REUG measurement, the position of the probe microphone was maintained for RECD measurement. RECD is another type of external-ear measurement; the main difference between RECD and REUG is that REUG is measured in the open-ear, while RECD is measured in the occluded-ear. In this experiment, the total RECD measurement reflects the total amount of gain (amplification) accumulated by a sound transduced via an insert-earphone that occludes the ear (as opposed to a free field speakers, as is the case during REUG measurement). External-ear amplification, in this case, occurs as a function of sound passing from the insert-earphone through the ear canal. The sound stimulus in a RECD measurement is a 55 dB-SPL pink noise (as measured inside a 1.4 cc coupler in 1/12 octave-band analysis) transduced to the ear by a ER-3A insert-earphone (Fig. 2). The insert earphone was placed such that its base was flush with the opening of the ear canal. As such, in this experiment, RECD refers to the difference between the total sound level observed inside the animal’s ear (recorded via probe microphone placed 2–3 mm from the tympanic membrane) and the known 55 dB-SPL pink noise signal measured inside of a coupler.

### Noise Exposure

Awake animals were exposed in their home cages (dimensions: 76 × 56 × 46 cm) to 89 dB-SPL (+ / − 2 dB) to 4 kHz octave-band noise (OBN) for 24 h. The OBN was generated using RPvdsEx software (Tucker Davis Technologies [TDT], Inc.), a TDT RM1 multifunctional processor, and a TDT PA4 programmable attenuator. Speakers (Fostex, FE127E) were individually mounted onto the center of the acoustically-transparent cage doors just above ear level (approximately 6 in. high) and above the free-feeding food container for each animal. The noise level was calibrated via sound level meter (Brüel & Kjær, Type 2250) at multiple points within the sound field (e.g., 3 in. from speaker; center of cage; back of cage). Throughout the exposure, animals had free access to food and water. Animal cages were cleared of obstructions such as enrichment items (toys, chews, and huts) prior to exposure.

## Statistical Analyses

Statistical analyses were conducted using IBM SPSS Statistics (v25) software package. Statistical significance level was interpreted as *p* < 0.05. Correlation strength was reported using Pearson’s correlation coefficient, the strength of which was interpreted according to “Rule of Thumb for Interpreting the Size of a Correlation Coefficient” found in Applied Statistics for the Behavioral Sciences [48]. The effect size of correlations was reported according to Cohen’s *f*^2^ effect size [49]. Ear canal volume (cm^3^) measurements were repeated and averaged for each animal. ABR threshold shifts (dB-nHL) were measured using three calculated PTAs: (1) average of all frequencies tested (“PTA-all”: 1, 2, 4, 8, 12, and 16 kHz), (2) average of mid-frequencies tested (“PTA-mid”: 2, 4, and 8 kHz), and (3) average of high frequencies tested (“PTA-high”: 8, 12, and 16 kHz). Statistical analyses were interpreted with caution, considering limited statistical power in a dataset of nine subjects. Homogeneity of variance in sex differences was analyzed by Levene’s test for all predictor variables and outcome measurements using both the group mean and group median. The only dependent variable that violated the homogeneity of variance assumption (Levene’s test *p* < 0.05) was 24 h threshold shift “PTA-mid” calculation. All other dependent variables passed the homogeneity of variance assumption (including the 24 h threshold shifts for “PTA-all” and “PTA-high,” and the 4-week threshold shift “PTA-all,” “PTA-mid,” and “PTA-high”). All independent variables (RECD, REUG, ear canal volume, and body weight) passed the homogeneity of variance assumption (Levene’s test *p* > 0.05). A one-way ANOVA was performed to assess the main effect of sex across all predictor variables and outcome measurements. Linear regression models were generated to test statistically significant correlations between EEA (including RECD and REUG) and ABR threshold shift, as well as correlations between body weight and ABR threshold shift.

## Results

### ABR Threshold Shift Differences: PTA and Sex

**Fig. 3:**
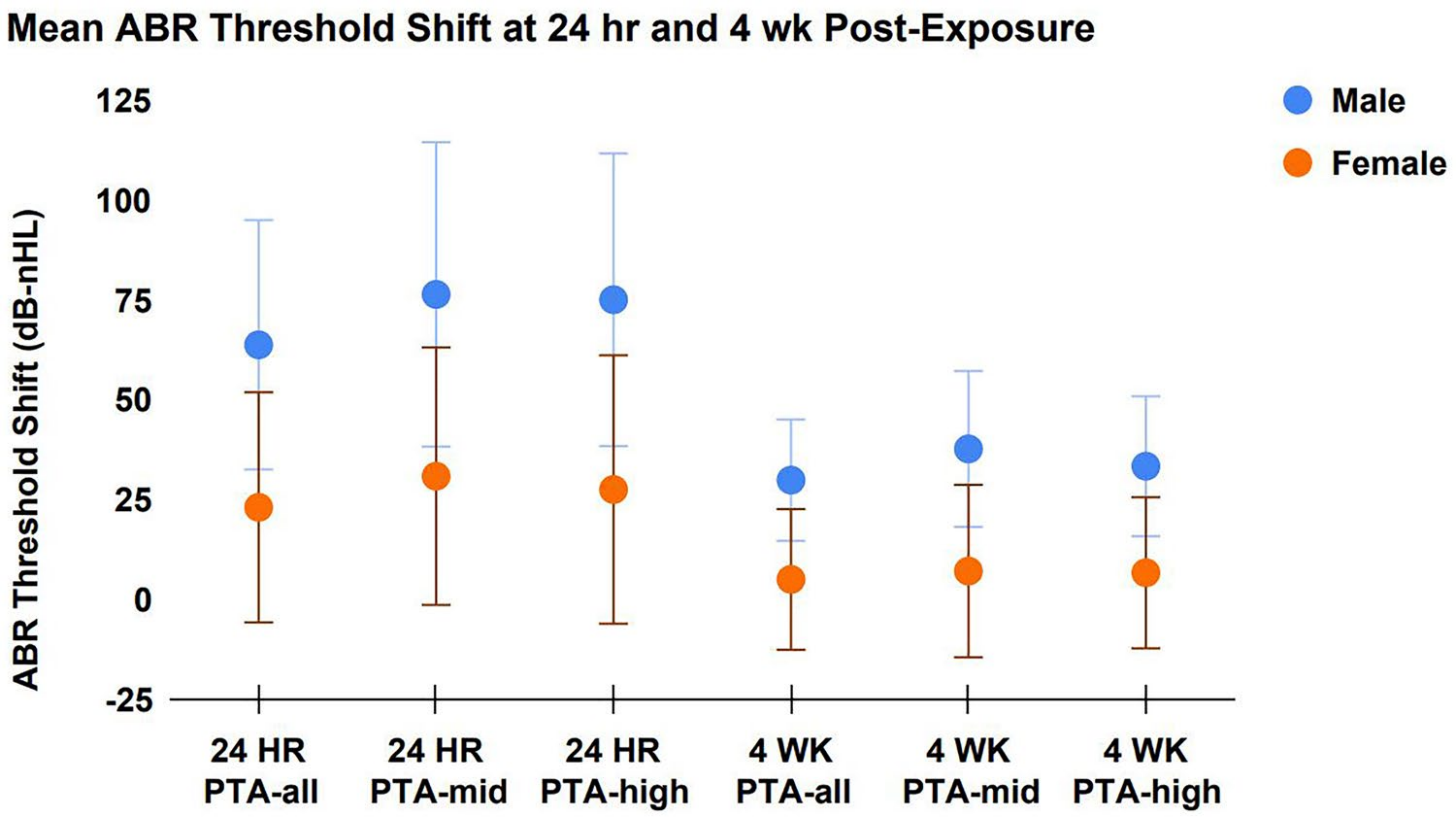
No statistically significant difference was observed in ABR threshold shift as a function of the various frequencies used to calculate each PTA, as described in the methods (i.e., PTA-all, PTA-mid, and PTA-high) (*p* = 0.78). As such, the PTA-all threshold shift measurement was used in all subsequent analyses. On average, male animals experienced significantly more ABR threshold shift than females animals at both 24-h and 4-week post-exposure (*p* = 0.01, *p* = 0.02; respectively)

A one-way ANOVA revealed no statistically significant differences in ABR threshold shift as a function of the PTA frequencies used to calculate the threshold (i.e., no statistically significant difference was observed between “PTA-all,” “PTA-mid,” and “PTA-high”). This was true of both the 24 h post-exposure measurement (*p* = 0.78) and 4-week post-exposure measurement (*p* = 0.86) (Fig. 3)) As such, the “PTA-all” calculation was used for all subsequent analyses. A one-way ANOVA revealed significant sex differences in both the 24-h and 4-week post-exposure ABR threshold measurements, with male animals experiencing more severe threshold shift than females animals (*p* = 0.01, *p* = 0.02; respectively) (Fig. 3).

### Sex Differences in Ear Canal Volume, RECD, REUG, and Body Weight

**Fig. 4:**
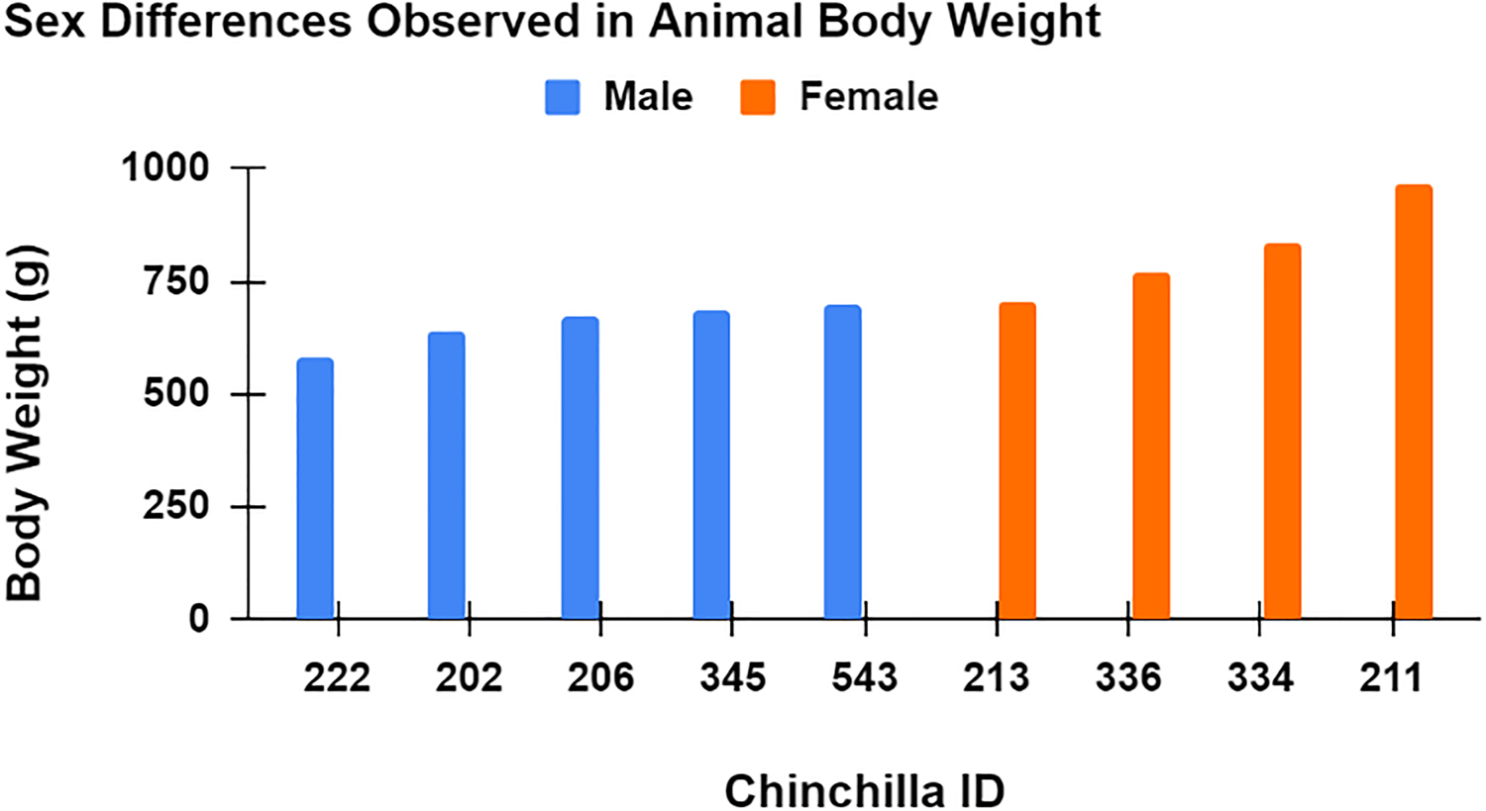
Body weight was consistently higher in female animals than males animals (female mean = 820 g, male mean = 654 g; *p* = 0.02)

**Fig. 5:**
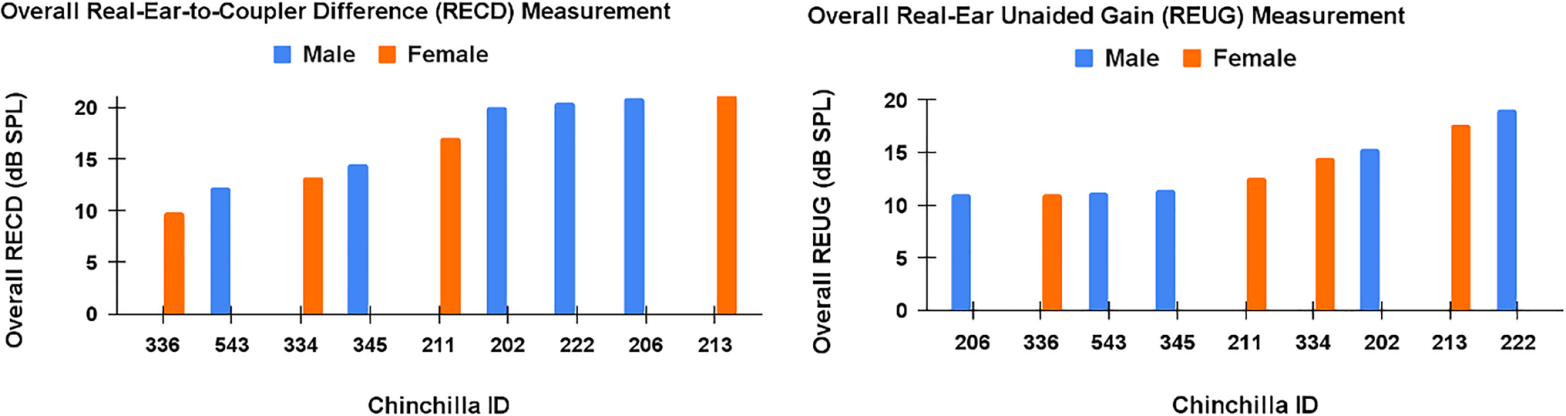
No statistically significant sex differences were observed in the RECD or REUG measurements (*p* = 0.60; *p* = 0.91, respectively). RECD ranged from 10–21 dB-SPL and REUG ranged from 11–19 dB-SPL

**Fig. 6:**
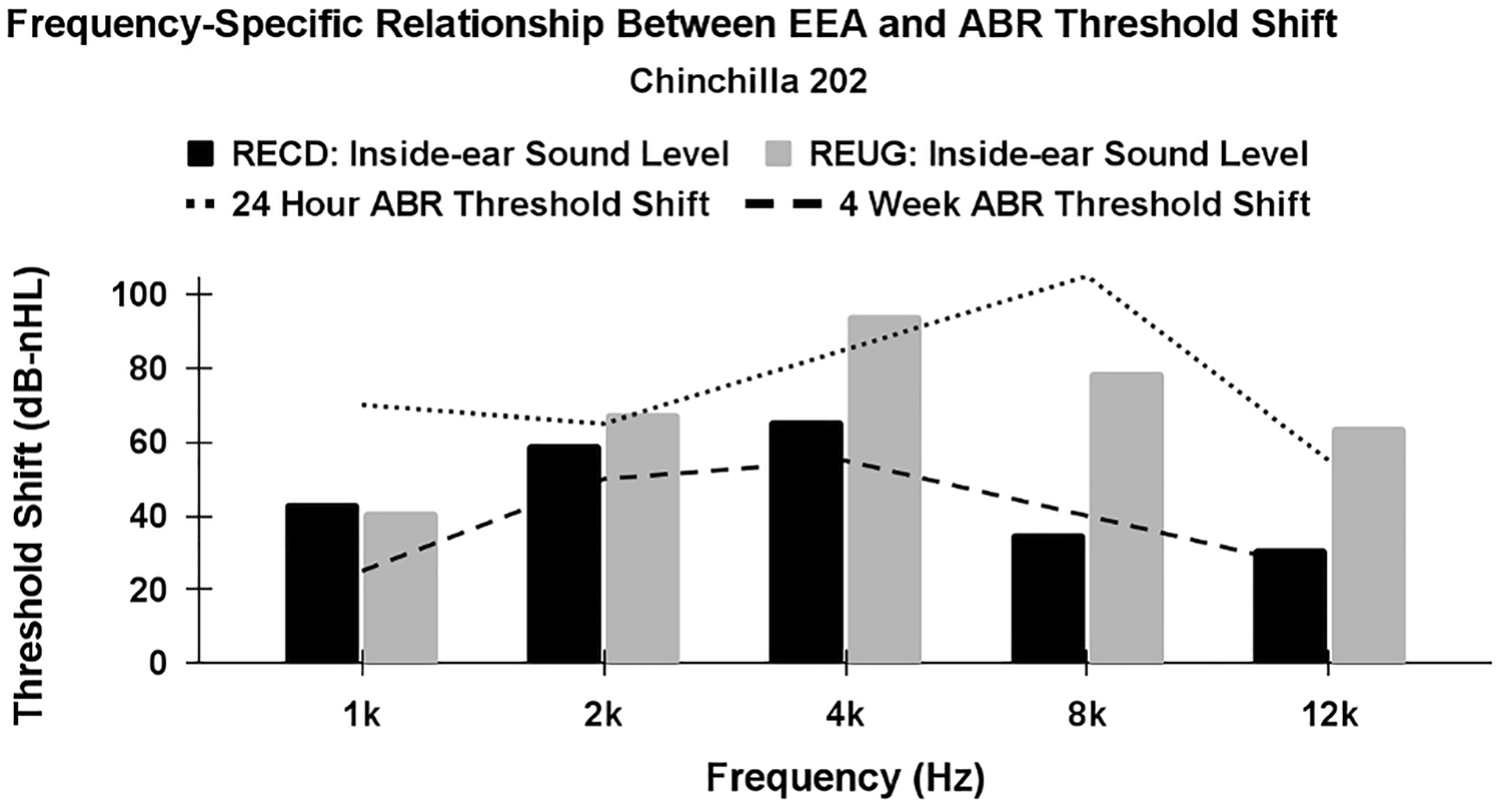
Shown in this figure is a representative animal (chinchilla 202; male) demonstrating the frequency-specific relationship across all animals between external-ear amplification (EEA) and subsequent ABR threshold shift at 24-h and 4-week post-exposure. Here, RECD and REUG are reported in their uncorrected difference (i.e., the true, in-ear microphone sound level during each measurement), in order to plot threshold shift and EEA together on the same scale. Peak resonances of the RECD and REUG were observed at approximately 4 kHz in each animal (slightly higher frequency than average adult human peak resonance; 2–4 kHz) and maximum, permanent (4 weeks) ABR threshold shift was observed at approximately 4 kHz in each animal (boldly dashed line)

Ear canal volume did not significantly differ (*p* = 0.90) between male animals (mean = 1.50 cm^3^) and female animals (mean = 1.51 cm^3^). Statistically significant sex differences were observed in body weight, with female animals weighing more than male animals on average (female mean = 820 g, male mean = 654 g; *p* = 0.02) (Fig. 4). A one-way ANOVA revealed no statistically significant sex differences in the RECD or REUG measurements (*p* = 0.60; *p* = 0.91, respectively) (Fig. 5). RECD ranged from 10–21 dB-SPL (male mean = 18 dB-SPL, female mean = 15 dB-SPL), and REUG ranged from 11–19 dB-SPL (female mean = 14 dB-SPL, male mean = 14 dB-SPL). Across male and female animals, peak resonance of the RECD and REUG was observed at approximately 4 kHz, and peak resonance was correlated with the ABR threshold frequency most severely worsened by the noise exposure (4 kHz)—at 4-week post-exposure (Fig. 6). The main purpose of evaluating peak-resonance of the animal’s EEA was to demonstrate that the chinchilla animal model serves as an effective model for translational external-ear measurement science, as peak resonance is closely aligned with that of adult humans (approximately 2–4 kHz).

### ABR Threshold Shift Susceptibility Differences: RECD, REUG, Ear Canal Volume, and Body Weight

**Fig. 7:**
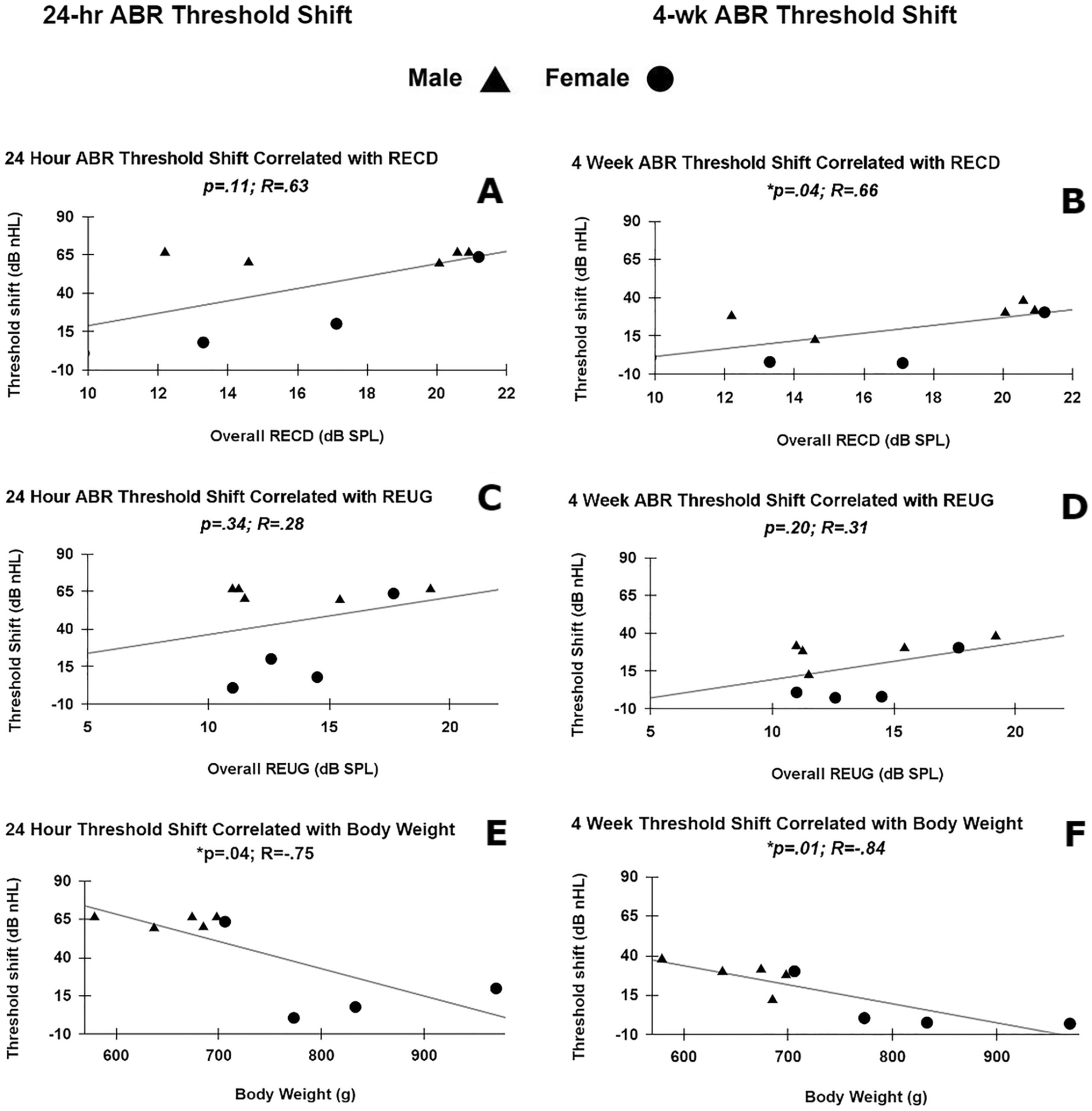
The left column panels **(A, C, E)** illustrate the relationships between RECD, REUG, body weight, and subsequent 24 h threshold shift, with each data point representing one animal. The right column panels **(B, D, F)** serve the same purpose, illustrated for the subsequent 4-week threshold shift. Statistical significance and correlation strength of each relationship are denoted inside each panel. The only statistically significant predictor of 24 h threshold shift susceptibility was body weight **(E)**, whereas permanent threshold shift (at 4-week) susceptibility was predicted by body weight **(F)** and RECD **(B)**. REUG did not serve as an effective predictor of threshold shift **(C, D)**

Ear canal volume was not correlated with threshold shift susceptibility at 24-h nor at 4-week post-exposure (Table 1). In the multiple regression model, lower body weight was the strongest predictor of increased NIHL susceptibility as measured by ABR threshold shift at 24 h (simple linear regression modeled *y* =  − 1.8*x + 175) and 4-week post-exposure (simple linear regression modeled *y* =  − 0.1*x + 109) (Fig. 7E, F) (Table 1). Higher EEA (RECD and REUG) was also correlated with increased threshold shift susceptibility at 4-week post-exposure, but not at 24-h post-exposure (Fig. 7A–D) (Table 1), with only RECD reaching statistical significance (simple linear regression modeled *y* = 2.65*x − 25.29) (Fig. 7B). Table 1 shows the correlations of RECD, REUG, ear canal volume, body weight, and subsequent ABR threshold shift as calculated using the “PTA-all” average threshold measurement method.

**Table 1:** Simultaneous linear regression models with independent variables RECD, REUG, ear canal volume, and body weight revealed statistically significant correlations between RECD, body weight, and ABR threshold shift at 4-week post-exposure, and between REUG and ABR threshold shift at 4-week post-exposure. Effects of RECD, REUG, ear canal volume, and body weight on ABR threshold shift

	**R**	**B**	**Std. error**	**Sig**
Model summary: **24 h PTA-all**	0.86		19.5	0.12
RECD	0.63	3.61	1.86	0.11
REUG	0.28	− 2.69	2.6	0.34
Ear canal volume	0.39	7.85	61.6	0.30
Body weight	− 0.75	− 0.15	0.06	*0.04
Model summary: **4 week PTA-all**	0.94		7.6	*0.01
RECD	0.66	2.23	0.81	*0.04
REUG	0.31	− 1.63	1.2	0.34
Ear canal volume	− 0.34	24.0	23.9	0.37
Body weight	− 0.84	− 0.11	0.03	*0.01

*R* Pearson correlation coefficient, *B* regression coefficient (slope), *Std. error* standard error of regression coefficient, *Sig* significance, *p* < 0.05 denoted with asterisk

## Summary and Conclusions

The purpose of this study was to test the hypothesis that higher external-ear amplification (EEA) would directly increase NIHL susceptibility (measured via ABR threshold shift) following a hazardous noise exposure in a chinchilla model. Nine chinchillas (5 male and 4 female) with normal hearing sensitivity were exposed to 4 kHz octave band noise for 24 hr at 89 dB SPL. Two types of EEA were measured prior to exposure, including real-ear-to-coupler-difference (RECD) (range = 10–21 dB-SPL; mean = 17 dB-SPL) and real-ear-unaided-gain (REUG) (range = 11–19 dB-SPL; mean = 14 dB-SPL). Of the frequencies sampled (1, 2, 4, 8, 12, and 16 kHz), peak resonance of the animal’s EEA was approximately 4 kHz. Sex differences in RECD and REUG were not statistically significant (*p* = 0.60; *p* = 0.91, respectively); however, males exhibited higher RECD (18 dB-SPL) than females (15 dB-SPL), on average. It was also of interest to investigate sex differences relative to threshold shift susceptibility, in addition to body weight differences (animal size) relative to threshold shift susceptibility. Male animals exhibited higher NIHL susceptibility than female animals (*p*=0.02). Animal body weight ranged from 579 to 970 g (mean = 728 g), with all four female animals weighing more than the five male animals (*p* = 0.02). 24-h post-exposure threshold shifts varied considerably across animals, from slight (0–10 dB HL) to profound (110 dB HL) (male mean = 64 dB HL; female mean = 23 dB HL). 4-week post-exposure threshold shifts were also variable, ranging from slight (0–10 dB HL) to moderate (50–60 dB HL) (male mean = 30 dB HL; female mean = 5 dB HL). Higher RECD was correlated with higher NIHL susceptibility (*R* = 0.66; *p* = 0.03), while REUG was not. Lower body weight was correlated with higher NIHL susceptibility (*R* =  − 0.84; *p* = 0.01). While the study was limited by a small sample size (*n* = 9), these preliminary results suggest that body size and EEA (a function of body size) may be an underlying driver of individual NIHL susceptibility differences, rather than true, genetic sex differences.

### Key Conclusions

- Male animals exhibited higher NIHL susceptibility than female animals (*p* = 0.02)
- All male animals exhibited lower body weight than female animals (*p* = 0.02)
- Lower body weight was correlated with higher NIHL susceptibility (*R* =  − 0.84; *p* = 0.01)
- Higher RECD was correlated with higher NIHL susceptibility (*R* = 0.66; *p* = 0.03)
- Male animals exhibited higher RECD than females on average; however, this was not a statistically significant difference in this dataset (*p* = 0.60)

## Discussion

In human research, a strong, well-known relationship exists between EEA and body size; higher EEA is correlated with smaller body size. Further, significant sex differences in body size are observed in most species. The results from this preliminary analysis suggest that, while sex may or may not genetically increase or decrease susceptibility to noise injury, it may also be a confounding variable obscuring the relationship between total external-ear amplification (EEA) and individual NIHL susceptibility, and/or body weight and individual NIHL susceptibility. In other words, while body size and biological sex are often correlated, there are exceptions, which would likely be reflected in EEA. As such, EEA may be a useful, overlooked predictor of individual NIHL susceptibility across sexes, from a simple mechanical, sound pressure level standpoint. The observed resonant frequencies and overall EEA gain in chinchillas in the present study reiterate that the chinchilla animal model serves as an effective model for translational external-ear measurement science, as peak resonance was closely aligned with that of humans (approximately 2–4 kHz) [21]. Koka et al. [50] and Jones et al. [51] previously established growth curves for chinchilla pinna dimensions and head circumference and studied their subsequent effect on directional transfer function of the open ear. The maximum gain observed in the aforementioned studies was approximately 10–15 dB in adult chinchillas; this variability is confirmed by the variability observed in the present study.

In this dataset, it also seems possible that there was a “critical point” observed in the ranges of RECD and body weight measurements when it came to NIHL susceptibility, as two distinct groups of animals can be clustered in the post-exposure ABR threshold shift data; those with RECD > 20 dB-SPL (Fig. 7B) and those with body weight < 750 g (Fig. 7F) were significantly more likely to experience more severe threshold shift at 4-week post-exposure (*p* = 0.01; *p* = 0.01, respectively). Notably, REUG was not correlated with NIHL susceptibility, while RECD was. We postulate that this could be a function of the variability in REUG being smaller than RECD; this may have decreased the likelihood of observing a linear relationship between an independent variable with low variability (REUG) and a dependent variable with high variability (subsequent threshold shift).

The niche within audiology that involves protecting an individual from recreational and occupational noise hazard has scientifically and logistically fallen behind the majority sector that prescribes amplification hearing loss. The current damage-risk criteria (DRC) formulaic approach to estimating an individual’s NIHL risk assumes that everyone who is exposed to the same level of noise for the same amount of time is equally at risk for NIHL. This assumption is analogous to suggesting that every individual with the same audiogram could be prescribed the same hearing aids. Such a suggestion would violate the gold standard amplification guidelines set forth by ASHA in 1998, which advise hearing aid programming adjustments for individual, external-ear amplification, in order to assure that hearing aid output is not under-amplified nor over-amplified.

We suggest borrowing this ASHA (1998) gold standard for application in hearing conservation and applying it to our understanding of a noise dose and individual NIHL risk hazard. A “noise dose” is a metric used to estimate the risk of NIHL from a sound exposure. For example, according to OSHA federal regulations (in which noise dose doubles with every additional 5 dB), increasing the sound level of an 85-dB workday exposure by 12 dB would increase an individual’s daily noise dose from 50% to 264%. According to NIOSH guidelines (in which noise dose doubles with every additional 3 dB), a 12 dB increase in sound level exposure would increase an individual’s workday noise dose from 100% to 1,600%. However, the current damage-risk criteria for NIHL does not account for the possibility that an individual’s EEA (above and beyond the free field sound level) could raise the total sound level exposure by 12 dB, thereby placing this individual at uniquely increased risk of NIHL. The 12 dB of total EEA variability that was observed in this animal model (REUG range = 11–19 dB) is consistent with the variability of total EEA observed in human models (5–19 dB) [14, 15]. The trends observed in this study (higher EEA correlated with increased post-exposure threshold shift) support the hypothesis that higher EEA is likely to directly and significantly influence individual NIHL susceptibility. This trend would be consistent with the current implementation of EEA knowledge in the sector of audiology that involves prescription of hearing aid amplification and verification based on individual EEA.

Separate from individual susceptibility and public health guidelines for hearing conservation, application of this knowledge could also be beneficial in clinical trial design for otoprotective agents in studies of NIHL—particularly in trials with small sample sizes. The authors suggest controlling for EEA by calibrating the noise exposure such that each subject receives the same noise dose inside the ear (as opposed to outside the ear, in the free field), as measured by the real-ear probe microphone technique used here. Milon and colleagues (2018) [52] have already suggested that NIHL studies should be separated by sex due to the observation that females appear to be less susceptible to NIHL than males (consistent with the present study’s results), which is consistent with prior experiments in humans [53] and animals [54]. We believe it is possible that female animals are, on average, less susceptible to NIHL as a function of sex; however, we would suggest that this decrease in susceptibility might actually be a direct function of EEA (which tends to be lower in female animals, which tend to be larger in body size), rather than a direct function of genetics and biologic sex. In addition to Milon et al.’s suggestion to account for sex in studies of NIHL susceptibility, we further suggest first controlling for the in-ear sound level of the noise exposure across subjects before searching for biological sex differences that might explain NIHL susceptibility.

## Data Availability

N/A.

## Code Availability

N/A.

## Abbreviations

ABR: Auditory brainstem response
ASHA: American Speech, Language, and Hearing Association
dB-A: A-weighted decibel level
dB-SPL: Unweighted decibel level
EEA: External-ear amplification
NIHL: Noise induced hearing loss
NIOSH: National Institute of Occupational Safety and Health
OBN: Octave band noise
OSHA: Occupational Safety and Health Administration
PTA: Pure tone average
PTS: Permanent threshold shift
RECD: Real-ear-to-coupler-difference
REUG: Real-ear unaided gain
TTS: Temporary threshold shift

## References

[1] Burns W, Robinson DW (1970) An investigation of the effects of occupational noise on hearing. In: Wolstenholme GEW, Knight J (eds) Sensorineural hearing loss: A Ciba Foundation symposium. Churchill, London, pp 177–192 10.1002/9780470719756.ch10 5210907

[2] Mills JH, Schulte BA, Boettcher FA, Dubno JR (2001) A comparison of age-related hearing loss and noise-induced hearing loss. In: Henderson D, Prasher D, Kopke R, Salvi RJ, Hamernik R (eds) Noise induced hearing loss: basic mechanisms, prevention and control. Noise Research Network, London, pp 497–511

[3] Passchier-Vermeer W (1973) Noise induced hearing loss from exposure to intermittent and varying noise. In: Proceedings of the International Congress on Noise as a Public Health Problem, May 13–18, Dubrovnik, Yugoslavia (Environmental Protection Agency, Washington DC), EPA 550/9–73–008:169–200

[4] Strasser H, Irle H, Legler R (2003). Temporary hearing threshold shifts and restitution after energy-equivalent exposures to industrial noise and classical music. Noise Health.

[5] Taylor W, Pearson J, Mair A, Burns W (1965). Study of noise and hearing in jute weaving. J Acoust Soc Am.

[6] Le Prell CG, Dell S, Hensley B, Hall III JW, Campbell KC, Antonelli PJ, Guire K (2012) Digital music exposure reliably induces temporary threshold shift in normal-hearing human subjects. Ear Hearing 33(6):e44–58 10.1097/AUD.0b013e31825f9d89 PMC3480981 22885407

[7] Ballachanda BB (1997). Theoretical and applied external ear acoustics. J Am Acad Audiol.

[8] Barnes J, Sabo RT, Coelho DH (2018). A novel method to measure the external auditory canal: normative data and practical implications. Am J Otolaryngol.

[9] Bastos BG, Ferrari DV, Blasc WQ (2012). Real ear unaided gain and its relation with the equivalent volume of the external and middle ear. Int Arch Otorhinolaryngol.

[10] Bingham K, Jenstad LM, Shahnaz N (2009). Longitudinal changes in real-ear to coupler difference measurements in infants. J Am Acad Audiol.

[11] Caiazzo AJ, Tonndorf J (1977). Ear canal resonance and temporary threshold shift. J Acoust Soc Am.

[12] Kuhn GF (1979). The pressure transformation from a diffuse sound field to the external ear and to the body and head surface. J Acoust Soc Am.

[13] Gerhardt KJ, Rodriguez GP, Hepler EL, Moul ML (1987). Ear canal volume and variability in the patterns of temporary threshold shifts. Ear Hear.

[14] Grinn S, Le Prell CG (2019). Noise-dose estimated with and without pre-cochlear amplification. J Acoust Soc Am.

[15] Grinn S, Le Prell CG (2021) Modeling individual noise-induced hearing loss risk with proxy metrics of external-ear amplification. Manuscript submitted, 2020 10.1121/10.0005061 34241484

[16] Hellström PA (1995). The relationship between sound transfer functions and hearing levels. Hear Res.

[17] Martin HC, Munro KJ, Lam MC (2001). Perforation of the tympanic membrane and its effect on the real-ear-to-coupler difference acoustic transform function. Br J Audiol.

[18] Mueller HG (2001). Probe microphone measurements: 20 years of progress. Trends Amplif.

[19] Pierson LL, Gerhardt KJ, Rodriguez GP, Yanke RB (1994). Relationship between outer ear resonance and permanent noise-induced hearing loss. Am J Otolaryngol.

[20] Rodriguez GP, Gerhardt KJ (1991). Influence of outer ear resonant frequency on patterns of temporary threshold shift. Ear Hear.

[21] Shaw EAG, Vaillancourt MM (1985). Transformation of sound pressure level from the free field to the eardrum presented in numerical form. J Acoust Soc Am.

[22] Sinclair ST, Beauchaine KL, Moodie KS, Feigin JA, Seewald RC, Stelmachowicz PG (1996). Repeatability of a real-ear-to-coupler difference measurement as a function of age. Am J Audiol.

[23] Ching TY, Dillon H (2003). Prescribing amplification for children: adult-equivalent hearing loss, real-ear aided gain, and NAL-NL1. Trends Amplif.

[24] Flanagan JL (1962). Speech analysis, synthesis, and perception.

[25] NIOSH (1998) Criteria for a recommended standard, occupational noise exposure. Department of Health and Human Services Publication No. 98–126 (National Institute on Occupational Safety and Health, Cincinnati, OH)

[26] OSHA (1983) 29 CFR 1910.95. Occupational noise exposure; hearing conservation amendment; Final Rule, effective 8 March 1983. U.S. Department of Labor. Occup Safety Health Admin

[27] Trevino M, Lobarinas E, Maulden AC, Heinz MG (2019). The chinchilla animal model for hearing science and noise-induced hearing loss. J Acoust Soc Am.

[28] Drescher DG, Eldredge DH (1974). Species differences in cochlear fatigue related to acoustics of outer and middle ears of guinea pig and chinchilla. J Acoust Soc Am.

[29] Dobie RA, Humes LE (2017). Commentary on the regulatory implications of noise-induced cochlear neuropathy. Int J Audiol.

[30] Quaranta A, Portalatini P, Henderson D (1998). Temporary and permanent threshold shift: an overview. Scand Audiol Suppl.

[31] Hamernik RP, Ahroon WA (1998). Interrupted noise exposures: threshold shift dynamics and permanent effects. The Journal of the Acoustical Society of America.

[32] Davis RI, Ahroon WA, Hamernik RP (1989). The relation among hearing loss, sensory cell loss and tuning characteristics in the chinchilla. Hear Res.

[33] Hamernik RP, Ahroon WA, Patterson JH, Qiu W (2002). Relations among early postexposure noise-induced threshold shifts and permanent threshold shifts in the chinchilla. J Acoust Soc Am.

[34] Patterson JH, Hamernik RP, Hargett CE, Ahroon WA (1993). An isohazard function for impulse noise. J Acoust Soc Am.

[35] Ward WD (1968). Temporary threshold shift and damage-risk criteria for intermittent noise exposures. J Acoust Soc Am.

[36] Masterson EA, Deddens JA, Themann CL, Bertke S, Calvert GM (2015). Trends in worker hearing loss by industry sector, 1981–2010. Am J Ind Med.

[37] Le TN, Straatman LV, Lea J, Westerberg B (2017). Current insights in noise-induced hearing loss: a literature review of the underlying mechanism, pathophysiology, asymmetry, and management options. J Otolaryngol Head Neck Surg.

[38] Ohlemiller KK, Wright JS, Heidbreder AF (2000). Vulnerability to noise-induced hearing loss in ‘middle-aged’ and young adult mice: a dose–response approach in CBA, C57BL, and BALB inbred strains. Hear Res.

[39] White PM (2019). Genetic susceptibility to hearing loss from noise exposure. Hear J.

[40] Wiener FM, Ross DA (1946). The pressure distribution in the auditory canal in a progressive sound field. J Acoust Soc Am.

[41] Ad Hoc Committee ASHA, on Hearing Aid Selection and Fitting.  (1998). Guidelines for hearing aid fitting for adults. Am J Audiol.

[42] Valente M (2006) Guideline for audiologic management of the adult patient. Audiology Online. https://www.audiologyonline.com/articles/guideline-for-audiologic-management-adult-966

[43] Shotland LI (1996). Dosimetry measurements using a probe tube microphone in the ear canal. J Acoust Soc Am.

[44] Dirks DD, Ahlstrom JB, Eisenberg LS (1996). Comparison of probe insertion methods on estimates of ear canal SPL. J Am Acad Audiol.

[45] Hawkins D (1991). Reliability of three types of probe tube microphone measurements. Hearing Instruments.

[46] Seewald RC, Feigin J, Stelmachowicz PG (1991) Hearing aid output limiting considerations for children. Pediatric Amplification: Proc Nat Conf 19–35

[47] Moodie KS, Seewald RC, Sinclair ST (1994). Procedure for predicting real-ear hearing aid performance in young children. Am J Audiol.

[48] Hinkle DE, Wiersma W, Jurs G (2003). Applied statistics for the behavioral sciences.

[49] Cohen J (1988) The effect size: r. In: Cohen J (ed) Statistical power analysis for the behavioral sciences. New York: Lawrence Erlbaum Associates, pp 77–83

[50] Koka K, Jones HG, Thornton JL, Lupo JE, Tollin DJ (2011). Sound pressure transformations by the head and pinnae of the adult chinchilla (Chinchilla lanigera). Hear Res.

[51] Jones HG, Koka K, Thornton JL, Tollin DJ (2011). Concurrent development of the head and pinnae and the acoustical cues to sound location in a precocious species, the chinchilla (Chinchilla lanigera). J Assoc Res Otolaryngol.

[52] Milon B, Mitra S, Song Y, Margulies Z, Casserly R, Drake V, Mong JA, Depireux DA, Hertzano R (2018) The impact of biological sex on the response to noise and otoprotective therapies against acoustic injury in mice. Biol Sex Differ 9(1):12 10.1186/s13293-018-0171-0 PMC5848513 29530094

[53] Szanto CS, Ionescu M (1983). Influence of age and sex on hearing threshold levels in workers exposed to different intensity levels of occupational noise. Audiology.

[54] McFadden SL, Zheng XY, Ding DL (2000). Conditioning-induced protection from impulse noise in female and male chinchillas. The Journal of the Acoustical Society of America.

